# The burden of illness in thyroid eye disease: current state of the evidence

**DOI:** 10.3389/fopht.2025.1565762

**Published:** 2025-04-17

**Authors:** Madhura A. Tamhankar, Syed Raza, Erika Brutsaert, Estefanía Urdániz, Yelena Vainilovich, Anne Heyes, Liesl Gildea, Marco Sales-Sanz

**Affiliations:** ^1^ Scheie Eye Institute, University of Pennsylvania, Philadelphia, PA, United States; ^2^ Global Health Economics and Outcome Research, argenx, Gerrards Cross, United Kingdom; ^3^ Clinical Development, argenx, Boston, MA, United States; ^4^ Translational & Clinical Sciences, argenx, Ghent, Belgium; ^5^ Clinical Development, argenx, Ghent, Belgium; ^6^ Value and Access, RTI Health Solutions, Manchester, United Kingdom; ^7^ Oculoplastic Surgery Unit, Ophthalmology, Hospital Universitario Ramón y Cajal, Madrid, Spain; ^8^ IMO Madrid, Grupo Miranza, Madrid, Spain

**Keywords:** thyroid eye disease, Graves ophthalmopathy, Graves orbitopathy, thyroid-associated ophthalmopathy, autoimmune disease, orbital condition

## Abstract

**Introduction:**

Thyroid eye disease (TED) is a disabling autoimmune condition characterized by proptosis and progressive orbital inflammation involving the extraocular muscles, orbital fat, and connective tissues. Clinical features include facial disfigurement, diplopia, dry eyes, and in severe cases, vision loss. Consequently, individuals with TED suffer significant physical and psychological burdens that impact their quality of life. Currently, there is no standardized definition or *International Classification of Diseases* code for TED, and the disease landscape remains incompletely understood; moreover, TED diagnostic criteria and treatment recommendations have not been thoroughly assessed across diverse populations. It is necessary to better understand the clinical, humanistic, and economic burden of TED and identify gaps in our knowledge to improve TED management and outcomes.

**Methods:**

To describe the current understanding of TED epidemiology, diagnosis, disease burden, and recent TED treatment guidelines, a targeted literature review was conducted, searching multiple databases using key words of specific search topics (i.e., TED; epidemiology, humanistic, economic, and clinical burden; treatment; and practice guidelines) for articles published between October 2013 and October 2023 in the United States, United Kingdom, and Europe (France, Germany, Italy, and Spain). Articles published between May 2014 and May 2024 describing diverse racial and sociodemographic presentations of TED were included.

**Results:**

TED is a complex disease with an array of risk factors, including thyroid dysfunction, thyroid-stimulating immunoglobin, smoking, and comorbid conditions. The natural history of TED is not clearly defined, and diagnosis is complicated due to the array of phenotypes and orbital symptoms observed. Although novel first-line treatments are available in select countries, there is an unmet need for improved treatments for moderate-to-severe and sight-threatening TED. Individuals with TED continue to experience poor health-related quality of life due to the clinical burden that TED imposes along with large healthcare resource utilization costs and treatment costs, and economic evaluation studies are limited. Importantly, there is still a need for studies that explore diverse populations and the impact of race and ethnicity on the disease landscape.

**Conclusion:**

TED remains an incompletely characterized disease with major knowledge gaps, particularly among historically underserved populations.

## 1 Introduction

Thyroid eye disease (TED) is a disabling condition that causes orbital inflammation, leading to proptosis, diplopia, and potential vision loss (1–3). It is one of the most frequently observed autoimmune inflammatory conditions of the orbit (4) and is prevalent in individuals with hyperthyroidism or those with a history of hyperthyroidism due to Graves’ disease (GD) (1). Although less common, TED also is observed in individuals with Hashimoto’s thyroiditis, or euthyroidism (5, 6). Although the precise pathogenesis of TED remains unclear, it generally is believed that affected individuals experience an acute inflammatory active phase, followed by resolution and quiescence of inflammation, during which proptosis and diplopia can persist (7–9). However, this paradigm of active and chronic disease recently has been brought into question (7, 8, 10). According to the European Group on Graves’ Orbitopathy (EUGOGO) classification system, TED is classified by severity into 3 groups: mild, moderate-to-severe, and sight threatening (5). Clinical features characteristic of mild TED includes mild proptosis, lid retraction, transient or absent diplopia, and dry eyes (5). Moderate-to-severe TED presents as exophthalmos ≥ 3 mm above normal for an individual’s gender and race, and lid retraction ≥2 mm, with intermittent or constant diplopia (5). Sight-threatening TED presents with dysthyroid optic neuropathy (DON) and/or exposure keratopathy (5). TED severity also is gauged by the clinical activity score (CAS), which may indicate the extent of active inflammation; according to EUGOGO, the American Thyroid Association (ATA), and the European Thyroid Association (ETA) guidelines on the management of TED, a CAS ≥3 is generally indicative of active TED (5, 11).

Treatment of TED should be considered following diagnosis to target the active phase of the disease and mitigate progression, with medical management recommendations based on clinical activity and severity assessments (5, 12–15). However, the diagnosis and classification of TED can be subjective due to inconsistency and lack of standardization in clinical and diagnostic tools (16, 17); indeed, no standardized definition or *International Classification of Diseases* (ICD) code exists for TED. The epidemiology, natural history, treatment guidelines, current management approaches, and disease burden of TED remain incompletely understood, especially among diverse and historically underserved populations. Moreover, there are no recommended differences in TED prevention and treatment modalities across racial and ethnic backgrounds, and diagnostic tools and treatments have not been thoroughly assessed in diverse populations (17, 18). To improve the recognition and management of TED, it is necessary to better understand the clinical, humanistic, and economic burden of TED and identify gaps in our understanding; to achieve this, we conducted a targeted landscape literature review of articles published in the United States (US), United Kingdom (UK), and Europe (France, Germany, Italy, and Spain).

## 2 Materials and methods

The targeted literature review searched PubMed, Embase, and the Cochrane Library databases for articles published within the last 10 years (October 2013 to October 2023) using a predefined search strategy; the topics and their respective key words used in the PubMed literature search are detailed in **Supplementary Table S1**. Comments, letters, editorials, and case reports were excluded in all searches (**Supplementary Table S1**). Subsequent searches for studies published within the last 10 years (May 2014 to May 2024) to capture equity and diversity in TED also were conducted in these databases using predefined search strategies (**Supplementary Table S2**). Articles were screened and selected according to title and abstract relevance, and additional articles were identified by examining the reference lists of the selected articles.

## 3 Results

**Figure 1:**
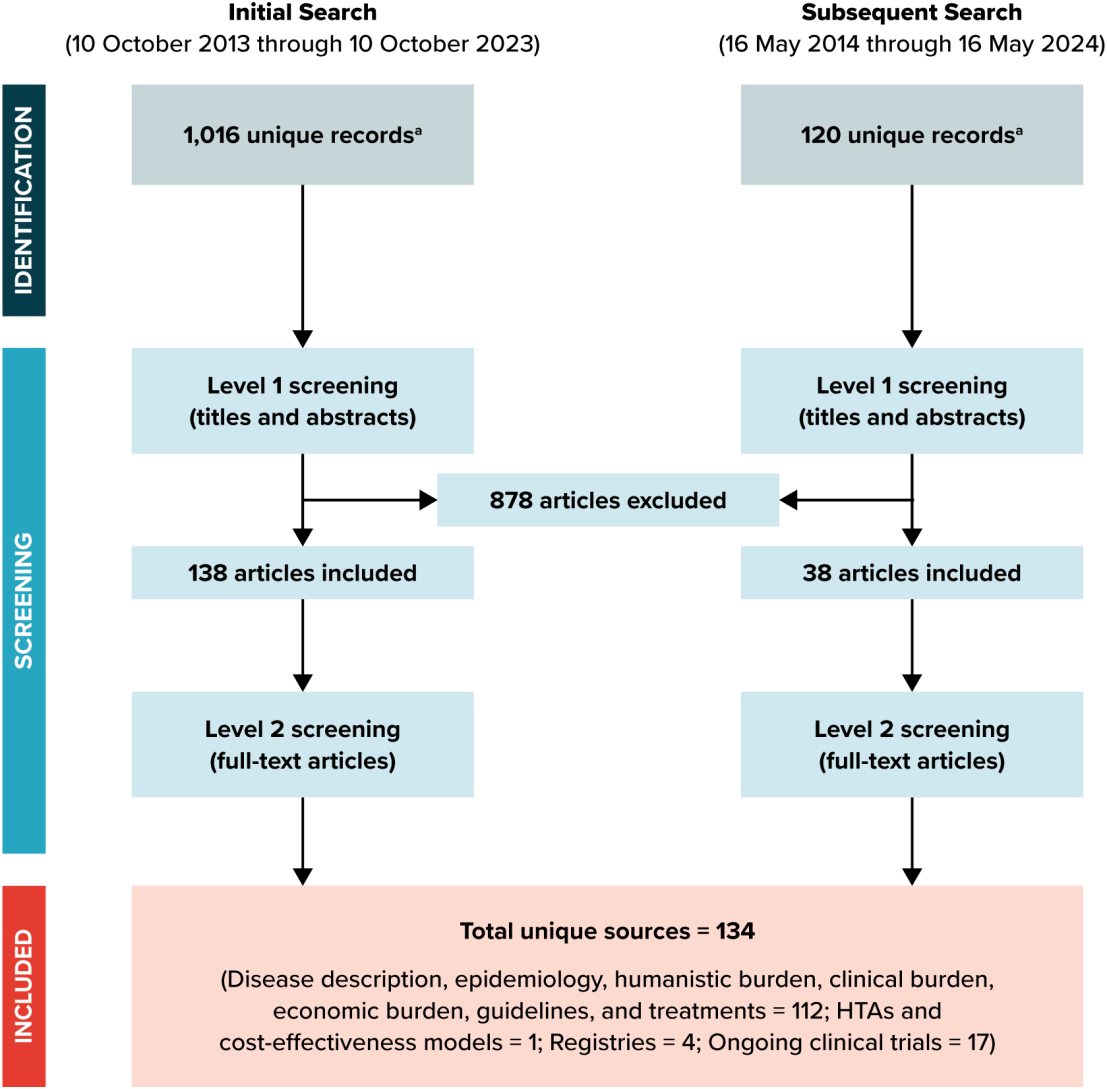
Flow diagram of study inclusion. HTA, health technology assessment. ^a^ All articles were obtained from PubMed, Embase, and the Cochrane Library databases, and duplicate articles were removed.

Out of 1,016 unique records identified during initial screening, 878 articles were irrelevant to the study objectives and were excluded in level 1 screening of titles/abstracts, resulting in the inclusion of 138 articles for full-text review during level 2 screening. Out of 120 unique records identified from subsequent searches targeting equity and diversity, 82 articles were excluded during level 1 screening, resulting in the inclusion of 38 articles for full-text review during level 2 screening. A total of 134 sources were selected for inclusion after conducting online desktop searches for health technology assessments and ongoing clinical trials (**Figure 1**).

### 3.1 Epidemiology

#### 3.1.1 Incidence and prevalence

Of the 134 included studies, few reported the incidence or prevalence of TED; however, a single large-scale, European-based study identified through EUGOGO reported that the prevalence of TED was approximately 10 per 10,000 people in 2017 (19). Additionally, a nationwide Danish study was identified that reported an overall mean annual incidence rate of 5 per 100,000 person-years in 2000-2018 (20). During the same period, rates were reported in the US on the basis of an analysis of the Intelligent Research in Sight (IRIS) patient registry database (21), with an overall observed prevalence of 0.09% during 2013-2018 (22). A widely cited estimate of the incidence of TED in the US, based on data from a representative county in Minnesota (Olmstead County) over a 15-year period (1976–1990), was 16 cases per 100,000 population per year for females and 2.9 cases per 100,000 population per year for males; notably, 100% of individuals with TED were White (6). TED is common among individuals with GD, with an estimated global prevalence of 40% (although prevalence rates vary across geographical regions), with the lowest estimated prevalence in North America (Oceania, 58%; Asia, 44%; Europe, 38%; North America, 27%) (23). Unfortunately, there are a limited number of studies comparing the incidence or prevalence of TED among different ethnic groups.

#### 3.1.2 Variance by age, sex, race, and ethnicity

TED most commonly affects females who are middle-aged (50-54 years), and a higher disease severity is found in older individuals (22, 24). Analysis of 2018 data from a large, US ambulatory surgery database estimated the mean age of individuals with severe TED that required eye surgery to be 56.2 years (25). The study also reported that surgery for TED was more common in women, representing 73% of all surgical patients (25). In alignment with this, analyses of patient data in the IRIS Registry (2013-2018) reported that the prevalence of TED was 3 times higher in women (0.12%) than in men (0.04%), a trend observed consistently across all age groups, races, and ethnicities (22). Additional US and European studies similarly estimate the incidence of TED to be higher in women than in men (3.3-16.0 cases vs. 0.9-2.9 cases per 100,000 person-years, respectively) (2, 6, 20). Notably, gender differences may be less pronounced in Asian populations (17).

There is a paucity of evidence comparing the prevalence of TED among different racial and ethnic groups, and studies show inconsistent findings. A recent analysis of the US IRIS database showed that more Black/African American (0.12%) and White (0.11%) people live with TED than Asian people (0.08%), and twice as many non-Hispanic people (0.10%) live with TED than Hispanic people (0.05%) (22). Interestingly, a study found that greater disease severity is associated with White individuals compared with Afro-Caribbean and Asian individuals (26), although 2 UK studies found no association between ethnicity and TED severity or between incidence of co-occurring autoimmune conditions and greater TED severity (27, 28). Further, peak prevalence age has been reported to differ by race and ethnicity (Asian and Hispanic, 30-39 years; Black/African American, 40-49 years; White and non-Hispanic, 50-59 years) (22). A small UK study (n=155) similarly reported that a lower prevalence of TED was found in individuals of Asian descent with GD (7.7%) compared with individuals of European descent with GD (42.0%) (17, 29) and, in another cross-sectional study (n=167), no significant differences in TED prevalence were found among Asian subpopulations with GD (40.0% in ethnically Indian individuals, 35.1% in ethnically Malay individuals, and 34.0% in ethnically Chinese individuals) (17, 30). In contrast, a meta-analysis reported the prevalence of TED to be higher in Asian (45%) than in White (37%) individuals with GD; however, these differences were not statistically significant (23).

#### 3.1.3 Risk factors

Thyroid dysfunction is a well-recognized risk factor for TED, with approximately 85% of individuals with TED developing Graves’ hyperthyroidism within the 18-month periods before or after hyperthyroidism onset (2). Thyroid-stimulating immunoglobin (TSI; otherwise known as thyroid-stimulating hormone or thyrotropin receptor autoantibodies) levels serve as a biomarker and as a risk factor of TED, where higher levels are strongly associated with TED severity (2, 3, 31, 32). In a European study, TSI activity was observed in 93.4% of individuals with TED (*P*<0.001), which demonstrates the potential for TSI levels to serve as a predictor of TED activity and severity (33). Treatment of GD with radioactive iodine has been associated with a rise in TSI levels, and precipitation or worsening of TED with radioactive iodine may be mediated by the spike in TSI levels (2, 3, 24, 34, 35).

Smoking is the largest, most important modifiable risk factor for TED (3), with smoking cessation being strongly recommended by EUGOGO guidelines (5). Current and past smokers have been reported to have significantly increased risk of developing TED compared with nonsmokers (odds ratio, 1.64 and 2.16, respectively; *P*<0.0001), and current and past smokers have an increased risk of developing TED-associated sight-threatening manifestations (22). Furthermore, past and current smoking has been associated with greater disease severity, a lower and slower response to immunosuppressive treatments, and greater likelihood of surgical intervention (3, 22, 36). One study found that orbital decompression and steroid treatment were required more frequently for smokers compared with nonsmokers—despite their younger age (24)—which underscores smoking as an independent risk factor for TED, even when adjusting for age. The mechanism through which smoking influences TED is not well understood, although it is proposed to involve increased oxygen-free radical generation and orbital hypoxia (2). Interventions aimed at helping individuals with TED with smoking cessation, such as tobacco cessation counseling, have proven effective; a retrospective cohort study found that 42.4% of individuals quit smoking following an ophthalmology consultation to advise against smoking (37).

Comorbid conditions including hypercholesterolemia, other autoimmune conditions such as type 1 diabetes mellitus, and oxidative stress such as that associated with smoking or systemic metabolic dysfunction are additionally reported to increase the risk of developing TED (2, 22, 38, 39). Other nonmodifiable risk factors include female sex and advancing age (6, 22, 24, 34, 40). Notably, TED has been associated with a higher mortality, particularly in males (41). Additionally, polymorphisms of genes related to immunity, thyroid function, adipogenesis, and DNA synthesis and repair have been found to increase the risk of TED (38). Race and ethnicity also may influence the risk of developing TED; however, the clinical evidence on disease severity by race and ethnicity is conflicting, as noted previously.

### 3.2 Natural history

The progression of TED traditionally has been depicted by using the biphasic Rundle’s curve model. However, the model is based on 2 observational studies with a low number of patients and has not been updated since its development (42–44). Additionally, the exact length of time it takes for TED to transition from the active phase to the inactive phase is unknown (2). Despite these drawbacks, it is useful to consider the 2 key phases of pathogenesis: an acute/active inflammatory phase (6-24 months) followed by a chronic/inactive noninflammatory phase (7, 8). Not all individuals with TED exhibit disease progression consistent with Rundle’s curve: different and unique phenotypes have been reported at different stages of the disease timeline (45).

In the acute phase, autoantibodies (including TSI) activate orbital fibroblasts, initiating a cascade of proinflammatory cytokines (i.e., interleukin [IL]-6, IL-12, IL-17, interferon gamma [IFN-γ], tumor necrosis factor alpha [TNF-α]), which contributes to orbital inflammation (3, 15, 16, 46–48). Additionally, cytokine upregulation leads to orbital fat deposition, as well as muscle and fat expansion (49, 50). This results in proptosis, diplopia, and periocular inflammation (16, 46).

During the chronic phase of TED, fibrotic changes can cause persistent proptosis and restrictive strabismus (16, 17). Individuals may experience relapses of TED during their lifetime, with or without therapy. Only 1 study identified in this review has assessed the natural progression of TED; a relapse rate of 15.7% was reported within the first 10 years following the initial episode of TED (9). Mild TED often remits spontaneously, but complete return to the pre-TED anatomical state rarely occurs in moderate-to-severe TED (2).

### 3.3 Diagnosis

Generally, TED diagnosis is based on the exclusion of confounding diagnoses (i.e., lymphoma, sarcoid, cellulitis), and some authors propose the inclusion of 2 of 3 diagnostic criteria categories (i.e., radiologic, laboratory, and clinical) (16, 34). Radiologic features, including tendon-sparing muscle enlargement and absence of orbital mass, on neurological imaging can help to establish the correct diagnoses in the majority of cases (16, 34, 51). Laboratory evidence of thyroid dysfunction, including presence of autoantibodies, can confirm the diagnosis (16, 34). Several validated clinical tools are frequently used to evaluate TED activity and severity (5). Considered the most validated measure, the CAS assesses disease activity by the presence of retrobulbar pain, pain on eye movements, eyelid edema and erythema, conjunctival injection, chemosis, and hypertrophy of plica and caruncle (5, 11, 16, 40). Other common classification tools include the globally used EUGOGO system to assess severity and the NOSPECS system (i.e., No physical signs or symptoms, Only signs, Soft tissue involvement, Proptosis, Extraocular muscle signs, Corneal involvement, and Sight loss) to assess progression (5, 52). The VISA system (i.e., Vision, Inflammation, Strabismus, and Appearance) and Bahn-Gorman score have also been used, but are not considered adequately validated, nor do they meet the criteria for objective clinician-reported outcomes (34, 53).

**Figure 2:**
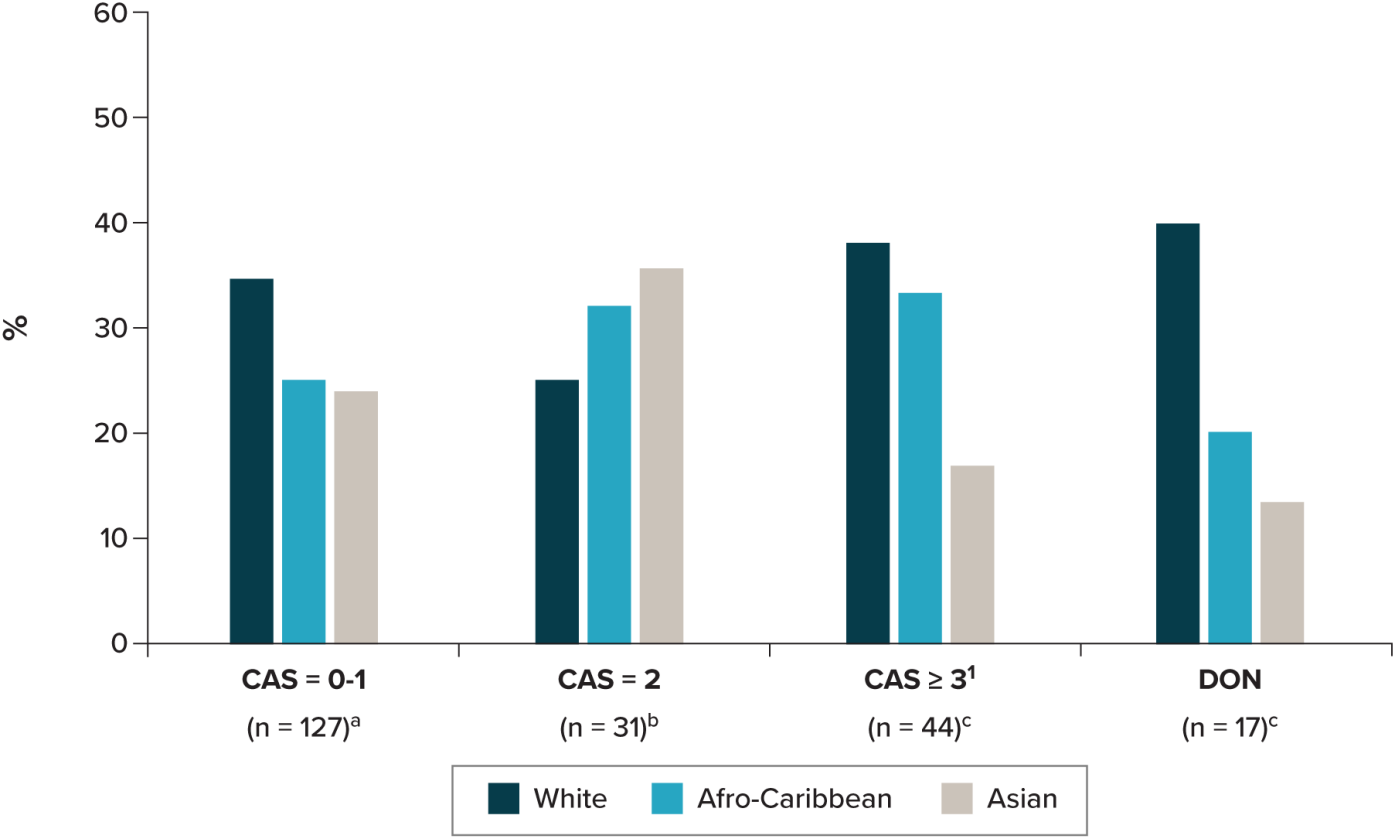
Disease activity at presentation by ethnicity. CAS, clinical activity score; DON, dysthyroid optic neuropathy. ^a^ Data missing, n=23. ^b^ Data missing, n=3. ^c^ Data missing, n=2. ^1^ Including non–sight-threatening TED. Source: Farag, Feeney (26).

Clinical presentation may influence the diagnosis of TED. Initial nonspecific symptoms of TED (e.g., eye redness, watery eyes, eye pain) may be misdiagnosed as chronic conjunctivitis, dry eyes, and/or ocular allergies and lead to incorrect treatment (54). Alternative causes of extraocular muscle enlargement (e.g., orbital mass lesions, inflammatory or neoplastic etiologies) can lead to proptosis and should be considered (55). Additionally, clinical presentation can be influenced by anatomical variations between different racial and ethnic groups (18); variation has been reported in the orbital wall (56), orbital floor (57), and exophthalmometry values (58). Proptosis is one of the most common presentations reported in Hispanic and Black populations compared with eyelid retraction (59), a presentation previously reported as common in White populations (60). Asian individuals have been reported to be at a higher risk of DON compared with White individuals due to anatomical differences (17). A study at 3 clinics in England reported White individuals were more likely to present with CAS ≥3 and DON compared with Afro-Caribbean and Asian individuals (**Figure 2**) (26). In contrast, a US study reported there to be no significant differences in TED severity between Black and White individuals (61).

### 3.4 Treatments

#### 3.4.1 Treatment guidelines

Early diagnosis and treatment of TED—including specialist care referrals, multidisciplinary approaches (e.g., including endocrinologists and ophthalmologists), lifestyle modification, and achieving euthyroidism—are key to limiting disease severity, improving clinical outcomes, and patient well-being (2, 11, 48, 62, 63). Treatment of TED is recommended immediately after diagnosis to target the acute inflammatory (active) phase; however, treatment is dependent on both the activity and severity of disease, as outlined by EUGOGO and the ATA and ETA consensus statements (5, 11, 13, 14). General treatment recommendations include the monitoring of thyroid function and maintaining euthyroidism, as well as smoking cessation, when applicable (5, 13, 15).

#### 3.4.2 Standard of care for TED

For cases of active, mild TED, EUGOGO guidelines recommend adopting general measures to control modifiable risk factors. This includes smoking cessation, dietary changes (i.e., adopting a diet low in salt and sugar), the use of topical lubricating eye drops, and, in some cases, a 6-month selenium supplementation for individuals residing in selenium-deficient areas (i.e., China, Russia, Europe) (5, 11, 13, 15, 54). It also is recommended that individuals be referred to centers that provide both endocrinological and ophthalmological expertise as well as expertise in the restoration of euthyroid status (54). If HRQOL is impaired, low-dose immunomodulatory therapy for active TED or rehabilitative surgery for inactive TED may be considered (5).

In active, moderate-to-severe TED, the initial treatment goal is to target activity and improve eye manifestations (i.e., proptosis and diplopia) (5). The ATA and ETA recommend intravenous (IV) glucocorticoids for individuals where severe proptosis/diplopia is absent and disease activity is the predominant feature (11). Similarly, EUGOGO guidelines recommend first-line IV glucocorticoids in alignment with global practice patterns (5, 54). Teprotumumab, an insulin-like growth factor-1 receptor approved in the US (64) and Brazil (65), is recommended as first-line treatment for those with proptosis and/or diplopia (11). Since 2023, teprotumumab has also been approved in the Kingdom of Saudi Arabia and Japan and is currently undergoing regulatory review in Australia, Europe, and Canada (66). Notably, a greater improvement in CAS and proptosis has been observed in clinical trial settings for participants who received teprotumumab compared with placebo, and early data reported in reviews (67, 68) suggested that teprotumumab can partially reverse the orbital tissue remodeling caused by TED (independent of active inflammation) and significantly reduced proptosis, strabismus, inflammation, and orbital soft tissue volume in individuals with chronic TED. In addition, several studies have shown that the insulin-like growth factor-1 receptor antagonist teprotumumab and the interleukin-6 receptor antagonist tocilizumab may be suitable for the treatments of DON, as they can reduce proptosis (69). Second-line treatments for moderate-to-severe and active TED include IV methylprednisolone, oral prednisone/prednisolone with cyclosporine or azathioprine (5), and orbital radiotherapy with glucocorticoids (5, 69, 70). Other monoclonal antibodies, including rituximab and tocilizumab, are also recommended as second-line treatments when glucocorticoid therapy has failed (5, 11).

Despite available treatment options, there remains an unmet need for improved moderate-to-severe TED treatments. Teprotumumab, although effective, is associated with adverse effects (e.g., hyperglycemia and hearing impairment/loss), so patient preferences and comorbidities need to be accounted for when balancing the benefits and risks of treatment (71–73). Furthermore, a relapse rate of 37% among patients treated with teprotumumab has been reported in 1 study by the US Food and Drug Administration (11). On the other hand, IV glucocorticoids do not consistently reduce long-term diplopia and proptosis; relapse rates of 20% to 40% previously have been reported (11). Due to numerous steroid-related adverse effects, patients require close monitoring (5, 13).

Sight-threatening TED is regarded as an emergency condition and requires immediate treatment with high-dose methylprednisolone (5, 54). If a patient is unresponsive to medical therapies, including those with DON, or has inactive TED, surgical intervention in the form of orbital decompression may be necessary to help relieve DON and corneal exposure keratopathy, as outlined by EUGOGO, ATA, and ETA guidelines (5, 11). Orbital decompression is one of the most performed surgeries for TED, followed by strabismus and eyelid surgery (7, 67). Diplopia may occur after orbital decompression and has been reported in 20% to 34% of patients (67, 74); it is hoped that the use of novel therapies like teprotumumab will reduce the number of patients requiring surgery (75).

#### 3.4.3 Treatment patterns

We identified a total of 18 ongoing clinical trials evaluating various therapies (**Supplementary Table S3**) and identified 1 health technology assessment on orbital irradiation from the National Institute for Health and Care Excellence. Practice patterns show a global trend of steroids as the preferred and most used treatment for active moderate-to-severe TED, with IV glucocorticoids cited as the most used agents (76, 77). Rituximab, tocilizumab, and teprotumumab were the top 3 monoclonal antibodies reported in articles from 2000 to May 2022 (78). Notably, monoclonal antibody use was not geographically uniform; tocilizumab and rituximab were prescribed primarily in Europe, with teprotumumab currently awaiting approval in Europe (78).

A cross-sectional survey of ATA and ETA members in 2021 found that European respondents reported a higher use of selenium (73%) for active, mild TED compared with North American respondents (32%) and respondents from other regions (24%) (79). For active, moderate-to-severe TED, there was a modest preference toward first-line treatment with teprotumumab among North American respondents (37%), whereas IV steroids were preferred among European respondents (73%) and respondents from other regions (42%), respectively (79).

In line with global treatment trends, a 2-part survey of the British Oculoplastic Surgery Society membership conducted from December 2016 to August 2017 found that most respondents used IV steroids (96%) (80). Similarly, a medical record review from December 2020 to January 2021 of US individuals with TED who were teprotumumab naive reported steroid use in the largest percentages of both individuals with long-term disease (>24 months) (70.6%) and those with short-term disease (≤24 months) (68.6%) (81). A 2018 US medical record review of individuals with moderate-to-severe TED (CAS ≥3) revealed that those with shorter TED duration were not treated as frequently with topical therapies as were those with longer TED durations, and steroid use was similar between those with longer and those with shorter TED durations, with an overall higher disease improvement among those with shorter TED duration (82). Studies in Europe support a general increase in use of surgery for the management of TED; surgical treatments were more frequently offered in 2012 than in 2000 (27.3% vs. 17%; *P*<0.05) among individuals referred to EUGOGO centers with inactive, mild disease (83). Similarly, data from the English national Hospital Episode Statistics from 1991 to 2011 found that the incidence of orbital decompression performed annually increased over the 2 decades; however, rates decreased after peaking in 2008 (84).

### 3.5 Clinical, humanistic, and economic burden

#### 3.5.1 Clinical burden

Individuals with TED experience visual dysfunction and facial disfigurement, which can substantially impact their HRQOL (85, 86). The clinical burden of TED tends to increase with disease activity and severity; a US retrospective review among individuals with moderate-to-severe TED found that those with inflammatory TED reported signs and symptoms more frequently than those with noninflammatory TED (87). These included dryness/grittiness, soft tissue edema, conjunctival redness, proptosis, excessive tearing, decreased vision, and pain with eye movement (87). Importantly, as proptosis, orbital changes, and vision dysfunction that developed during the initial progressive inflammatory phase could persist chronically, it was further suggested that moderate-to-severe TED be considered a symptomatic and chronic disease, regardless of the inflammatory state (87). In a separate study, 75.2% of individuals reported having diplopia, which profoundly interferes with various activities of daily living, driving, and working (88). Certain comorbid conditions are commonly reported in association with TED; for example, in 1 study, 16.7% of individuals with TED reported having an additional autoimmune disease (e.g., vitiligo, chronic autoimmune gastritis, rheumatoid arthritis, polymyalgia rheumatica) (89), and data from the National Institutes of Health revealed that 29% of TED cases had glaucoma compared with 6% of non-TED controls (90).

#### 3.5.2 Humanistic burden and HRQOL

**Figure 3:**
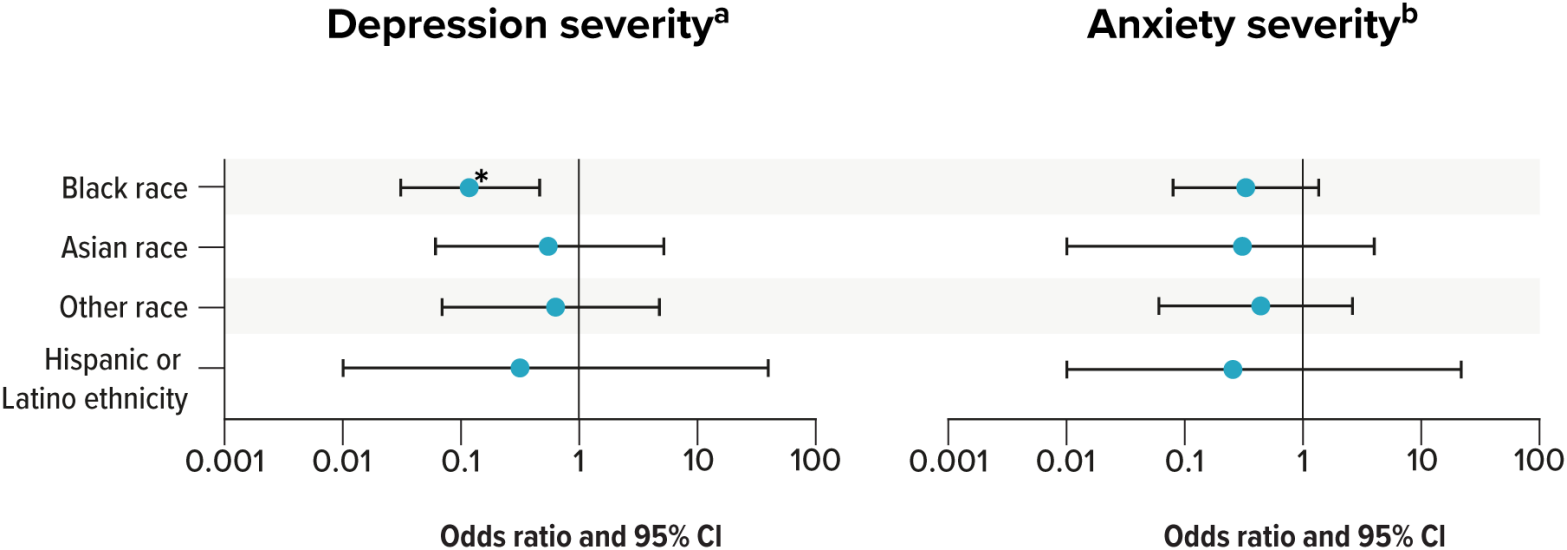
Predictors of increased severity of depression and anxiety in TED. * P ≤ 0.05. CI, confidence interval; GAD-7 = General Anxiety Disorder-7; PHQ-9, Patient Health Questionnaire-9; TED, thyroid eye disease. ^a^ PHQ-9 scores are shown for depression severity. ^b^ GAD-7 scores are shown for anxiety severity. Source: Lee, Radha-Saseendrakumar (97).

The physical impact of TED has been documented, with 1 patient survey revealing that 69% of individuals with TED suffer from physical symptoms that are present throughout all phases of the disease (91). Additionally, TED is emotionally distressing and significantly affects social functioning, leading to neuropsychiatric disorders (92). Increasing disease severity and activity is associated with reduced HRQOL, as confirmed by treating physicians (87, 93). Notably, inaccuracy in TED diagnosis can cause distress to individuals, which arises from (1) the lack of standardized diagnostic tools and (2) a reliance on subjective and potentially inconsistently reported clinical signs and symptoms (94). These factors can lead to treatment delays and risk vision loss. Many studies have reported that individuals with TED experience poorer HRQOL compared with those with other chronic conditions (e.g., heart failure, emphysema, and diabetes) (92, 95). One study reported overall mental health condition rates of 36% to 37% for individuals with noninflammatory and inflammatory TED, with anxiety and depression reported in 26% to 28% and 17% to 19% of both populations, respectively (87). Utility values (cardinal values that capture preferences for various health outcomes in an individual) for individuals with active, moderate-to-severe TED have previously been defined in a qualitative US study (96). It reported that individuals with moderate-to-severe TED suffer greater disutility than those with mild TED. The most severe disease state was associated with the lowest mean utility value of 0.30 (95% confidence interval [CI], 0.24-0.36), whereas the least severe disease state was associated with the highest mean utility value of 0.60 (95% CI, 0.54-0.67) (96). Importantly, another US-based study found that individuals of all races and ethnicities with TED are significantly more likely to experience clinical depression compared with control populations (97). Black race was found to be a protective factor for severe depression, as marked by a lower Patient Health Questionnaire-9 Items (PHQ-9) score (odds ratio, 0.12; 95% CI, 0.03-0.45; *P*=0.002) (**Figure 3**), despite depression prevalence reportedly being higher for Black race (97).

A retrospective chart review revealed that US individuals with inflammatory TED (N=307) suffer greater impairment to their HRQOL than individuals with noninflammatory TED (N=281), with psychological well-being reported as the most impaired item (**Table 1**) (87). However, results from a US online survey completed by ophthalmologists and endocrinologists revealed higher disease activity and severity were associated with a greater HRQOL impact, specifically in orbital pain, visual disturbances (including diplopia), and orbitofacial structural changes (93). Through use of the Graves’ Orbitopathy-Specific Quality of Life (GO-QOL) questionnaire, HRQOL has been shown to improve following treatment (i.e., immunotherapy, radiotherapy, and surgery) (98–101). For example, in 1 prospective follow-up study of participants who had undergone immunotherapy with IV methylprednisolone, quality-of-life subscales on the GO-QOL questionnaire significantly increased post therapy, with sustained improvement for 6 months (98). Similarly, in a study among participants who received orbital radiotherapy, GO-QOL visual functioning scores improved significantly by 6 months, and Appearance scores increased through 12 months post therapy (100). The effect of different surgical procedures also has been assessed using the GO-QOL, with substantial improvements observed in HRQOL, including greater perceived effects on appearance than visual function (101). However, orbital decompression surgery was reported to significantly improve the HRQOL in individuals with inactive, moderate-to-severe TED, as assessed by the GO-QOL, including a statistically significant increase in mean values of visual functioning (99).

**Table 1:** Impact of TED on the HRQOL of patients with inflammatory and noninflammatory disease.

Domain of daily functioning	Inflammatory TED (CAS ≥3)	Noninflammatory TED (CAS = 0 or 1)
Overall QOL impact	4.7	3.6^*^
Work/school	4.1	3.1*
Social	4.5	3.4^*^
Daily activities	4.4	3.3*
Driving	4.2	3.1*
Psychological well-being	4.6	3.6^*^

^*^ *P*<0.001.

CAS, clinical activity score; HRQOL, health-related quality of life; QOL, quality of life; TED, thyroid eye disease. Mean data for the QOL impact are shown and are scored on a 7-point scale, where 1 = not at all impaired and 7 = extremely impaired.

Source: Wang, Padnick-Silver (87).

#### 3.5.3 Economic burden

**Figure 4:**
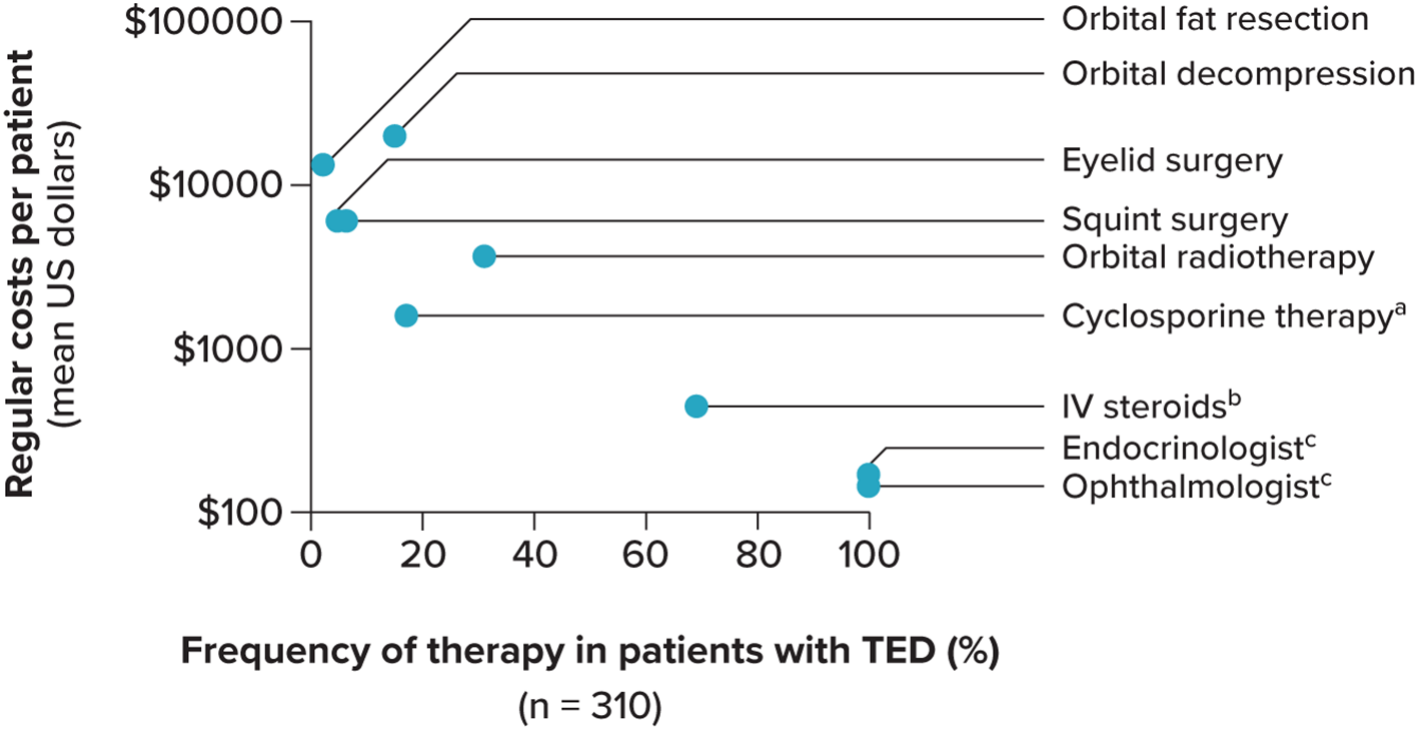
Direct costs in patients With TED. IV, intravenous; TED, thyroid eye disease. Standard deviation not shown. ^a^ Body weight adapted. ^b^ Methylprednisolone, 6 weeks of 500 mg weekly, followed by 6 weeks of 250 mg weekly. ^c^ Outpatient treatment. Source: Ponto, Merkesdal (103).

Only 3 economic studies on TED were identified: 2 US retrospective studies (25, 102) and 1 German cross-sectional study (103). Hospitalizations, emergency visits, and treatment costs were found to be the major drivers of direct costs associated with the burden of TED treatment (25, 103). Surgery had a significant economic impact on individuals with severe TED in the US, with total charges for TED surgery exceeding $43.5 million annually; the average charge for each surgical encounter was $21,875 (25). The German cross-sectional study conducted from 2005 to 2009 estimated the total direct costs associated with the treatment of TED within the total German population to be $200,122,640 per year (103). As shown in **Figure 4**, 100% of individuals sought outpatient treatment by an ophthalmologist (mean cost, $138; standard deviation [SD], $8.8) or visited an endocrinologist (mean cost, $147; SD, $8.7); individuals also commonly received IV steroids (69%; mean cost, $438; SD, $2.3) and orbital radiotherapy (31%; mean cost, $3,573; SD, $0). Other studies that did not primarily focus on the economic impact of TED revealed the high expense associated with TED treatments—specifically, those recommended as first-line treatments. The ATA and ETA consensus statement reported that the total cost of a course of teprotumumab is $357,997 for a 75-kg patient, which is significantly higher than the cost of second-line treatments, such as rituximab ($19,636 [€4,308] for the largest dose available for administration) and tocilizumab ($14,519 [€4,266]) (11).

Aside from direct costs, TED is associated with indirect costs for individuals, such as time off from work, not returning to work after sickness absence, work role limitations, unemployment, and being on disability benefits (102, 103). A 2020 US retrospective study of 100 individuals revealed that 14% of those with inactive, chronic TED reported disabilities/unemployment and sought an average of 19.7 (SD, 31.7) TED-related physician visits in the year before study participation (102). The cross-sectional study identified diplopia as the principal predictor for work disability, and individuals with optic neuropathy were nearly twice as likely to be work disabled as individuals without a compression of the optic nerve (60% versus 33%; *P*=0.075) (103). The mean duration of sick leave was 22.3 (SD, 60.8) days per year, which was higher than the German average (11.6 days/year) and significantly correlated with disease severity (103). Overall, indirect costs for individuals with TED were predicted to average between approximately $1.7 and $3.5 billion per year (103).

## 4 Discussion

Our targeted literature review summarizes the current landscape for TED, including its epidemiology, disease profile, diagnosis, treatment guidelines, and burden of illness in the US, UK, and Europe. Despite having an impactful disease burden and an association with excess morbidity and mortality, TED remains an incompletely characterized disease with major knowledge gaps in each of these areas, particularly among diverse and historically underserved populations. Though multifactorial, these evidence gaps are likely influenced by the lack of a standardized definition or ICD code for TED.

The paucity of recent studies in the US or Europe evaluating the epidemiology of TED—as well as the heterogeneity of methodology and results between different studies impedes the estimation of current TED incidence and prevalence rates. There is presently a reliance on outdated data in the literature, with data from 1994 from Olmstead County, Minnesota, US, being widely referenced (6). Although some studies have reported that age, gender, race, and ethnicity influence TED epidemiology, evidence for this is limited; indeed, the Olmstead County population assessed in 1994 was 100% White. Like the pathogenesis of TED, the role of age, sex, race, and ethnicity on the severity and risk of disease development remains poorly understood. Further research on the natural history of TED will help inform treatment pathways that may prevent complications needing surgery and be tailored to each patient based on individual clinical manifestations or disease duration. Similarly, it is important to further explore how social determinants of health can help stakeholders understand the economic impacts on lower-resourced populations.

The diagnosis of TED can be challenging due to inconsistently reported signs and symptoms and varies due to the lack of standardization in clinical parameters and diagnostic tools, several of which remain to be validated or thoroughly evaluated in diverse populations. Without standardized assessments, it is also difficult to evaluate and compare treatment outcomes across randomized clinical trials. Currently, teprotumumab is the only pharmaceutical treatment approved in the US for the treatment of TED, with additional global approvals pending, including recent approval in Japan (66). There are a total of 18 ongoing clinical trials evaluating alternative treatments for TED. Thus, despite the availability of multiple treatment options for TED, there is still a lack of evidence on the importance of many interventions—especially novel therapies (53, 104)—on long-term patient outcomes (11, 13, 14, 54, 70). As supported by robust comparison studies with current first-line treatments, there remains an unmet need for novel treatments that address issues of access, tolerability, and durability of response. Further research is warranted to understand the impact of earlier interventions, which may influence the progression of the disease and subsequently improve long-term outcomes. Notably, most studies have been conducted on White populations—even though race and ethnicity are reported to influence the impact of TED—although there have been inconsistent findings. This inconsistency is likely driven by differences in diagnosis due to disease presentation and referral to care. Additionally, there are no recommended differences in TED treatment guidelines across racial and ethnic populations, despite known disease variations. Establishment of diagnostic and assessment standards that take into consideration diverse populations and the effect of race and ethnicity on TED may enhance the development and clinical implementation of targeted therapies, leading to improved outcomes for individuals with TED.

Although we found minimal longitudinal data on HRQOL in individuals with TED, the identified studies generally supported HRQOL to be significantly reduced by TED. In contrast, improved function with surgery and changes in appearance were inconsistently associated with improvement in HRQOL, which warrants further investigation. Evidence of the economic burden of TED also was profoundly limited in this study, with only 3 studies identified in the literature. Moreover, in these studies, evaluation of the costs of TED were limited to the US and Germany only and did not include newer treatments, such as teprotumumab. Further studies are required to understand the economic burden of the disease in other countries to account for different costs, social systems, and treatment competitors.

This landscape review has highlighted the distribution of TED risk and burden among various ethnic groups across multiple countries, underscoring the clinical, humanistic, and economic burdens faced by individuals with TED and providing a comprehensive review of the current TED standards of care. Limitations, including those inherent in literature reviews, should be considered. Because there is no ICD code assigned for TED, ICD codes were not used to search for publications and the data captured in this review may be incomplete. Similarly, limited studies were obtained during our analyses for several areas of interest, which limited our ability to draw conclusions and make comparisons. For instance, only 3 studies reporting the economic burden of TED were identified, and no information was identified on the costs of surgery in mild versus moderate-to-severe disease. The geographical scope of this targeted literature review was limited to literature published in the US, UK, and Europe, and therefore, our findings may not be globally representative. However, taken together, the results of this landscape review suggest that TED is a complex disease influenced by demographic factors that impact the diagnosis, treatment, and burden of illness in people with TED. Future research efforts considering the presently identified key evidence gaps will improve the therapeutic landscape for individuals with TED.
