# Supplementary material for: The burden of illness in thyroid eye disease: current state of the evidence
Source: Front Ophthalmol (Lausanne). 2025 Apr 17;5:1565762. doi: 10.3389/fopht.2025.1565762 (PMC12075187; doi:10.3389/fopht.2025.1565762)
Supplement: Supplementary file 1 [file Table1.docx]

# Supplementary Material

Table S1. PubMed Literature Search Strategy for TED

| Search no. | Search terms | Hits |
| --- | --- | --- |
| Thyroid eye disease | | |
| #1 | “Graves Ophthalmopathy”[Majr] OR “graves ophthalmopath*”[Title] OR “thyroid-associated ophthalmopathy”[Title] OR “thyroid-associated ophthalmopathies”[Title] OR “thyroid eye disease”[Title] OR “thyroid eye diseases”[Title] OR “dysthyroid ophthalmopath*”[Title] OR “Graves eye disease”[Title] OR “Graves orbitopath*”[Title] OR “myopathic ophthalmopath*”[Title] OR “congestive ophthalmopath*”[Title] OR “edematous ophthalmopath*”[Title] OR “infiltrative ophthalmopath*”[Title] OR “graves’ ophthalmopath*”[Title] OR “Graves’ eye disease”[Title] OR “Graves’ orbitopath*”[Title] OR “grave’s ophthalmopath*”[Title] OR “Grave’s eye disease”[Title] OR “Grave’s orbitopath*”[Title] | 2,134 |
| Epidemiology | | |
| #2 | #1 AND (“Graves Ophthalmopathy/epidemiology”[Majr] OR “Graves Ophthalmopathy/etiology”[Majr] OR “Epidemiology”[Majr] OR “Incidence”[Majr] OR “Prevalence”[Majr] OR “Morbidity”[Majr] OR “Risk Factors”[Majr] OR “Age Distribution”[Mesh] OR “Sex Distribution”[Mesh] OR epidemiol*[Title] OR inciden*[Title] OR prevalen*[Title] OR morbidit*[Title] OR risk*[Title] OR demographic*[Title] OR “age distribution”[Title/Abstract] OR “sex distribution”[Title/Abstract] OR “race distribution”[Title/Abstract] OR “ethnicity distribution”[Title/Abstract]) | 478 |
| Humanistic burden | |  |
| #3 | #1 AND (“Quality of Life”[Majr] OR “Self Report”[Mesh] OR “Activities of Daily Living”[Mesh] OR “Patient Satisfaction”[Mesh] OR “Patient Preference”[Mesh] OR “Caregivers”[Mesh] OR “Health Status”[Mesh] OR “Functional Status”[Mesh] OR “Social Stigma”[Mesh] OR “Mental Health”[Mesh] OR “Emotions”[Mesh] OR burden*[Title] OR “quality of life”[Title] OR caregiver*[Title/Abstract] OR “care giver*”[Title/Abstract] OR “life quality”[Title/Abstract] OR QoL[Title/Abstract] OR “GO-QOL”[Title/Abstract] OR “TED-QOL”[Title/Abstract] OR hrql[Title/Abstract] OR hrqol[Title/Abstract] OR “SF-36”[Title/Abstract] OR sf36[Title/Abstract] OR sf12[Title/Abstract] OR “SF-12”[Title/Abstract] OR sf24[Title/Abstract] OR “SF-24”[Title/Abstract] OR “well being”[Title/Abstract] OR wellbeing[Title/Abstract] OR “functional status”[Title/Abstract] OR “functional dependence”[Title/Abstract] OR “functional independence”[Title/Abstract] OR “self report*”[Title/Abstract] OR “patient report*”[Title/Abstract] OR “activities of daily living”[Title/Abstract] OR “activity of daily living”[Title/Abstract] OR “daily living activity”[Title/Abstract] OR “daily living activities”[Title/Abstract] OR “activity of daily life”[Title/Abstract] OR “activities of daily life”[Title/Abstract] OR “daily life activity”[Title/Abstract] OR “daily life activities”[Title/Abstract] OR “physical function*”[Title/Abstract] OR “patient satisfaction”[Title/Abstract] OR “treatment satisfaction”[Title/Abstract] OR “health status”[Title/Abstract] OR “health state”[Title/Abstract] OR “patient preference*”[Title/Abstract] OR “patient’s preference*”[Title/Abstract] OR “patients’ preference*”[Title/Abstract] OR “patient perspective*”[Title/Abstract] OR “patient’s perspective*”[Title/Abstract] OR “patients’ perspective*”[Title/Abstract] OR “patient experience*”[Title/Abstract] OR “patient’s experience*”[Title/Abstract] OR “patients’ experience*”[Title/Abstract] OR “unmet need*”[Title/Abstract] OR “EQ-5D”[Title/Abstract] OR EQ5D[Title/Abstract] OR “social stigma*”[Title/Abstract] OR “mental health”[Title/Abstract]) | 162 |
| Economic burden | | |
| #4 | #1 AND (“Graves Ophthalmopathy/economics”[Mesh] OR “Health Care Costs”[Mesh] OR “Economics”[Mesh] OR “Economics, Hospital”[Mesh] OR “Economics, Medical”[Mesh] OR “Economics, Pharmaceutical”[Mesh] OR “Economics, Nursing”[Mesh] OR “Insurance, Disability”[Mesh] OR “Health Expenditures”[Mesh] OR “Cost of Illness”[Mesh] OR “Cost-Benefit Analysis”[Mesh] OR “Direct Service Costs”[Mesh] OR “Absenteeism”[Mesh] OR “Efficiency”[Mesh] OR “Length of Stay”[Mesh] OR “Health Resources/economics”[Mesh] OR “Fees and Charges”[Mesh] OR “Budgets”[Mesh] OR “Costs and Cost Analysis”[Mesh] OR “Quality-Adjusted Life Years”[Mesh] OR “Presenteeism”[Mesh] OR costs[Title/Abstract] OR cost[Title/Abstract] OR costly[Title/Abstract] OR economic*[Title/Abstract] OR pharmacoeconomic*[Title/Abstract] OR fiscal[Title/Abstract] OR fee[Title/abstract] OR fees[Title/Abstract] OR expenditure*[Title/Abstract] OR budget*[Title/Abstract] OR disabilit*[Title/Abstract] OR “resource use”[Title/Abstract] OR “resource utilization”[Title/Abstract] OR “resource utilisation”[Title/Abstract] OR “length of stay”[Title/Abstract] OR “quality adjusted life year”[Title/Abstract] OR “quality adjusted life years”[Title/Abstract] OR QALY*[Title/Abstract] OR absenteeism[Title/Abstract] OR productiv*[Title/Abstract] OR “lost work”[Title/Abstract] OR “work loss”[Title/Abstract] OR “missed work”[Title/Abstract] OR “sick day*”[Title/Abstract] OR “illness day*”[Title/Abstract] OR presenteeism[Title/Abstract] OR “health disparit*”[Title/Abstract]) | 39 |
| Clinical burden | |  |
| #5 | #1 AND (“Graves Ophthalmopathy/complications”[Majr] OR “Graves Ophthalmopathy/mortality”[Majr] OR “Comorbidity”[Majr] OR “Disease Progression”[Majr] OR “Mortality”[Majr] OR “Severity of Illness Index”[Majr] OR “Diagnostic Errors”[Majr] OR “Delayed Diagnosis”[Majr] OR “Patient Readmission”[Majr] OR complicat*[Title] OR sequelae[Title] OR comorbidit*[Title] OR multimorbidit*[Title] OR “co-morbidit*”[Title] OR “multi-morbidit*”[Title] OR “disease progression”[Title] OR “disease exacerbation”[Title] OR “progression of disease”[Title] OR mortalit*[Title] OR death*[Title] OR fatal*[Title] OR “clinical activity score”[Title] OR CAS[Title] OR exophthalmos[Title] OR proptosis[Title] OR proptoses[Title] OR diplopia*[Title] OR “double vision”[Title] OR polyopsia*[Title] OR VISA[Title] OR “vision inflammation strabismus and appearance”[Title] OR “severity of illness index”[Title] OR “illness index severity”[Title] OR “illness index severities”[Title] OR “diagnostic error*”[Title] OR misdiagnosis[Title] OR misdiagnoses[Title] OR “diagnostic blind spot”[Title] OR “diagnostic blind spots”[Title] OR “missed diagnosis”[Title] OR “delayed diagnosis”[Title] OR “delayed diagnoses”[Title] OR “late diagnosis”[Title] OR “late diagnoses”[Title] OR readmission*[Title] OR rehospitalization*[Title] OR rehospitalisation*[Title] OR “re-hospitalization*”[Title] OR “re-hospitalisation*”[Title]) | 257 |
| Treatment | | |
| #6 | #1 AND ((“Graves Ophthalmopathy/drug therapy”[Majr] OR “Graves Ophthalmopathy/therapy”[Majr] OR “Graves Ophthalmopathy/surgery”[Majr] OR “Graves Ophthalmopathy/radiotherapy”[Majr] OR “Drug Therapy”[Majr] OR “Therapeutics”[Majr] OR “Biological Products”[Majr] OR “Radiotherapy”[Majr] OR “teprotumumab”[Supplementary Concept] OR “belimumab”[Supplementary Concept] OR “Rituximab”[Mesh] OR “tocilizumab”[Supplementary Concept] OR “3-(8-amino-1-(2-phenylquinolin-7-yl)imidazo(1,5-a)pyrazin-3-yl)-1-methylcyclobutanol”[Supplementary Concept] OR “Sirolimus”[Mesh] OR “Drug-Related Side Effects and Adverse Reactions”[Majr] OR “Immunosuppressive Agents”[Majr] OR “Hydroxymethylglutaryl-CoA Reductase Inhibitors”[Majr] OR “Immunoglobulins, Intravenous”[Majr] OR “Plasma Exchange”[Majr] OR treat*[Title] OR therap*[Title] OR “monoclonal antibod*”[Title] OR “biological product*”[Title] OR biopharmaceutical*[Title] OR biologic[Title] OR biologics[Title] OR “biological drug*”[Title] OR “biological medicine*”[Title] OR teprotumumab[Title] OR tepezza[Title] OR “RV-001”[Title] OR RV001[Title] OR “R-1507”[Title] OR R1507[Title] OR RO4858696[Title] OR “RO4858696-000”[Title] OR “RO-4858696”[Title] OR “RO-4858696000”[Title] OR “RO-4858696-000”[Title] OR belimumab[Title] OR benlysta[Title] OR GSK1550188[Title] OR “GSK-1550188”[Title] OR “LymphoStat-B”[Title] OR HGS1006[Title] OR “HGS-1006”[Title] OR BEL114333[Title] OR “BEL-114333”[Title] OR rituximab[Title] OR Rituxan[Title] OR GP2013[Title] OR “IDEC-C2B8”[Title] OR mabthera[Title] OR tocilizumab[Title] OR atlizumab[Title] OR actemra[Title] OR roactemra[Title] OR “RHPM-1”[Title] OR “RO-4877533”[Title] OR “R-1569”[Title] OR “RG-1569”[Title] OR “MSB-11456”[Title] OR MSB11456[Title] OR batoclimab[Title] OR HBM9161[Title] OR “IMVT-1401”[Title] OR vunakizumab[Title] OR “SHR-1314”[Title] OR SHR1314[Title] OR “VRDN-001”[Title] OR linsitinib[Title] OR “OSI-906”[Title] OR OSI906[Title] OR “FcRn inhibitor*”[Title] OR “FcRn antagonist*”[Title] OR “IL6R inhibitor*”[Title] OR “anti-IL6R”[Title] OR “anti-IGF-1R”[Title] OR “IGF-1R inhibitor*”[Title] OR “IGF-1R antagonist*”[Title] OR “K1-70”[Title] OR IVIg[Title] OR “intravenous antibod*”[Title] OR “intravenous immune globulin”[Title] OR “intravenous immunoglobulin*”[Title] OR “intravenous immune globulins”[Title] OR “intravenous IG”[Title] OR “IV immunoglobulin*”[Title] OR “Flebogamma DIF”[Title] OR Gamunex[Title] OR “Globulin-N”[Title] OR Intraglobin[Title] OR Gammagard[Title] OR Gamimune[Title] OR Gamimmune[Title] OR Privigen[Title] OR “modified immune globulin”[Title] OR sandoglobulin[Title] OR venoglobulin[Title] OR venimmune[Title] OR iveegam[Title] OR alphaglobin[Title] OR endobulin[Title] OR Gammonativ[Title] OR PLEX[Title] OR “plasma exchange*”[Title] OR sirolimus[Title] OR rapamune[Title] OR rapamycin[Title] OR “AY-22989”[Title] OR “AY 22-989”[Title] OR I2190A[Title] OR “I-2190A”[Title] OR surger*[Title] OR surgical[Title] OR radiotherap*[Title] OR cyclosteroid*[Title] OR “steroidal compound”[Title] OR steroids[Title] OR steroid[Title] OR “anti-inflammat*”[Title] OR antiinflammat*[Title] OR immunosuppressant*[Title] OR “immunosuppressive agent*”[Title] OR NSISTs[Title] OR “nonsteroidal immunosuppressive therapy”[Title] OR “nonsteroidal immunosuppressive therapies”[Title] OR “non-steroidal immunosuppressive therapy”[Title] OR “non-steroidal immunosuppressive therapies”[Title] OR statin*[Title] OR “hydroxymethylglutaryl CoA reductase inhibitor”[Title] OR “hydroxymethylglutaryl CoA reductase inhibitors”[Title] OR “HMG COA reductase inhibitor”[Title] OR “HMG COA reductase inhibitors”[Title] OR “hydroxymethylglutaryl-coenzyme A inhibitor”[Title] OR “hydroxymethylglutaryl-coenzyme A inhibitors”[Title] OR “hydroxymethylglutaryl-CoA inhibitor”[Title] OR “hydroxymethylglutaryl-CoA inhibitors”[Title] OR “side effect*”[Title] OR “adverse event*”[Title] OR “adverse reaction*”[Title] OR “drug reaction*”[Title] OR “drug toxicit*”[Title] OR “drug event*”[Title] OR “Immunosuppressive Agents”[Pharmacological Action] OR “Hydroxymethylglutaryl-CoA Reductase Inhibitors”[Pharmacological Action]) AND (systematic[sb] OR review*[Title] OR “systematic review*”[Title/Abstract] OR SLR[Title/Abstract] OR “literature review*”[Title/Abstract] OR “Review”[Publication Type] OR “Systematic Review”[Publication Type])) | 190 |
| Practice patterns and guidelines | | |
| #7 | #1 AND (“Practice Patterns, Physicians’”[Majr] OR “Practice Guidelines as Topic”[Mesh] OR “Practice Guideline”[Publication Type] OR “treatment pattern*”[Title/Abstract] OR “practice pattern*”[Title/Abstract] OR “current treatment*”[Title/Abstract] OR “treatment options”[Title/Abstract] OR “best practice*”[Title/Abstract] OR “treatment paradigm*”[Title/Abstract] OR challeng*[Title] OR guideline*[Title]) | 69 |
| Exclusions | | |
| #8 | “Animals”[Mesh] NOT “Humans”[Mesh] | 1,157,886 |
| #9 | “Comment”[Publication Type] OR “Letter”[Publication Type] OR “Editorial”[Publication Type] OR “Case reports”[Publication type] OR “case stud*”[Title] OR “case report*”[Title] OR “case series”[Title] OR “case histor*”[Title] | 1,502,060 |
| Total | | |
| #10 | (#2 OR #3 OR #4 OR #5 OR #6 OR #7) NOT (#8 OR #9) | **737** |

TED = thyroid eye disease.

Note: Search conducted October 2023.
