# Supplementary material for: The burden of illness in thyroid eye disease: current state of the evidence
Source: Front Ophthalmol (Lausanne). 2025 Apr 17;5:1565762. doi: 10.3389/fopht.2025.1565762 (PMC12075187; doi:10.3389/fopht.2025.1565762)
Supplement: Supplementary file 2 [file Table2.docx]

# Supplementary Material

Table S2. Subsequent PubMed Literature Search Strategy for TED

| Search no. | Search terms | Hits |
| --- | --- | --- |
| Thyroid eye disease | | |
| #1 | “Graves Ophthalmopathy”[Mesh] OR “graves ophthalmopath*”[Title/Abstract] OR “thyroid-associated ophthalmopathy”[Title/Abstract] OR “thyroid-associated ophthalmopathies”[Title/Abstract] OR “thyroid eye disease”[Title/Abstract] OR “thyroid eye diseases”[Title/Abstract] OR “dysthyroid ophthalmopath*”[Title/Abstract] OR “Graves eye disease*”[Title/Abstract] OR “Graves orbitopath*”[Title/Abstract] OR “myopathic ophthalmopath*”[Title/Abstract] OR “congestive ophthalmopath*”[Title/Abstract] OR “edematous ophthalmopath*”[Title/Abstract] OR “infiltrative ophthalmopath*”[Title/Abstract] OR “dysthyroid eye disease*”[Title/Abstract] OR “dysthyroid orbitopath*”[Title/Abstract] OR “endocrine orbitopath*”[Title/Abstract] OR “graves’ eye disease*”[Title/Abstract] OR “graves’ ophthalmopath*”[Title/Abstract] OR “graves’ orbitopath*”[Title/Abstract] OR “ophthalmic graves disease*”[Title/Abstract] OR “ophthalmic graves’ disease*”[Title/Abstract] OR “thyroid associated eye disease*”[Title/Abstract] OR “thyroid associated orbitopath*”[Title/Abstract] OR “thyroid ophthalmopath*”[Title/Abstract] OR “thyroid orbitopath*”[Title/Abstract] OR “thyroid related orbitopath*”[Title/Abstract] OR “endocrine ophthalmopath*”[Title/Abstract] OR “Thyroid-related eye”[Title/Abstract] | 6,827 |
| Health disparities – general terms | | |
| #2 | “Health Inequities”[Mesh] OR “health inequit*”[Title/Abstract] OR “health inequalit*”[Title/Abstract] OR “health care inequalit*”[Title/Abstract] OR “healthcare inequalit*”[Title/Abstract] OR “health care inequit*”[Title/Abstract] OR “healthcare inequit*”[Title/Abstract] OR “health disparit*”[Title/Abstract] OR “health status disparit*”[Title/Abstract] OR “health status inequalit*”[Title/Abstract] OR “health status inequit*”[Title/Abstract] OR “Healthcare Disparities”[Mesh] OR “healthcare disparit*”[Title/Abstract] OR “health care disparit*”[Title/Abstract] OR “disparities in health*”[Title/Abstract] OR “disparity in health*”[Title/Abstract] OR “Social Determinants of Health”[Mesh] OR “social determinant*”[Title/Abstract] OR “structural determinants of health”[Title/Abstract] OR “health structural determinant*”[Title/Abstract] OR “social determining factor*”[Title/Abstract] OR “social factors determining health”[Title/Abstract] OR “social health determinant*”[Title/Abstract] OR “Health Equity”[Mesh] OR “health equit*”[Title/Abstract] OR “health status equit*”[Title/Abstract] OR “health status equalit*”[Title/Abstract] OR “health care equalit*”[Title/Abstract] OR “healthcare equalit*”[Title/Abstract] OR “health care equit*”[Title/Abstract] OR “healthcare equit*”[Title/Abstract] | 98,325 |
| #3 | #1 AND #2 | 4 |
| Right to health | |  |
| #4 | “Right to Health”[Mesh] OR “health care right*”[Title/Abstract] OR “health right*”[Title/Abstract] OR “healthcare right*”[Title/Abstract] OR “right to accessible health*”[Title/Abstract] OR “right to health*”[Title/Abstract] | 2,696 |
| #5 | #1 AND #4 | 0 |
| Place or state of residence/sociodemographic factors | | |
| #6 | “sociodemographic factors”[Mesh] OR ((sociodemographic*[Title/Abstract] OR “socio demographic*”[Title/Abstract]) AND (factor*[Title/Abstract] OR determinant*[Title/Abstract])) OR “Residence Characteristics”[Mesh] OR “Residence Characteristic*”[Title/Abstract] OR “Environment Design”[Mesh] OR “Environment Design*”[Title/Abstract] OR “Healthy Place*”[Title/Abstract] OR “environmental plan*”[Title/Abstract] OR “Urban Population”[Mesh] OR “Urban Population*”[Title/Abstract] OR “urban health services”[Mesh] OR “Urban Health”[Mesh] OR “urban health*”[Title/Abstract] OR “rural population”[Mesh] OR “rural population*”[Title/Abstract] OR “rural health services”[Mesh] OR “rural health”[Mesh] OR “rural health*”[Title/Abstract] OR “rural area*”[Title/Abstract] OR “community resources”[Mesh] OR “community resource*”[Title/Abstract] OR “Health Services Geographic Access*”[Title/Abstract] | 351,058 |
| #7 | #1 AND #6 | 7 |
| Socioeconomic status | |  |
| #8 | “Socioeconomic Factors”[Mesh:NoExp] OR “Social Welfare”[Mesh:NoExp] OR “Social Class”[Mesh] OR “Poverty”[Mesh] OR “Income”[Mesh] OR “Economic Status”[Mesh] OR “working poor”[Mesh] OR “social discrimination”[Mesh] OR “social marginalization”[Mesh] OR “social segregation”[Mesh] OR “medically uninsured”[Mesh] OR “social class*”[Title/Abstract] OR “social status”[Title/Abstract] OR “social position*”[Title/Abstract] OR “Social background*”[Title/Abstract] OR “Social circumstance*”[Title/Abstract] OR “socio economic*”[Title/Abstract] OR “socioeconomic*”[Title/Abstract] OR disadvantaged[Title/Abstract] OR impoverished[Title/Abstract] OR poverty[Title/Abstract] OR “economic level*”[Title/Abstract] OR “economic status”[Title/Abstract] OR “assets index*”[Title/Abstract] OR income*[Title/Abstract] OR “social disparit*”[Title/Abstract] OR “social factor*”[Title/Abstract] OR “social inequal*”[Title/Abstract] OR “social inequit*”[Title/Abstract] OR “working poor*”[Title/Abstract] OR “uninsured”[Title/Abstract] OR discrimination[Title/Abstract] OR marginalization[Title/Abstract] OR segregation[Title/Abstract] | 811,429 |
| #9 | #1 AND #8 | 37 |
| Gender differences | | |
| #10 | “Gender Identity”[Mesh] OR “gender identit*”[Title/Abstract] OR “gender differen*”[Title/Abstract] OR “Gender inequal*”[Title/Abstract] OR “Gender inequit*”[Title/Abstract] OR “gender disparit*”[Title/Abstract] OR “sex disparit*”[Title/Abstract] OR “sex differenc*”[Title/Abstract] OR “sex role*”[Title/Abstract] OR “woman role*”[Title/Abstract] OR “women role*”[Title/Abstract] OR “man role*”[Title/Abstract] OR “men role*”[Title/Abstract] OR “gender role*”[Title/Abstract] | 127,642 |
| #11 | #1 AND #10 | 11 |
| Ethnicity | | |
| #12 | “Health Services, Indigenous”[Mesh] OR “Black People”[Mesh] OR “American Indian or Alaska Native”[Mesh] OR apartheid[Mesh] OR “Asian People”[Mesh] OR “Ethnicity”[Mesh] OR “Hispanic or Latino”[Mesh] OR “indians, north American”[Mesh] OR “mexican americans”[Mesh] OR “Oceanians”[Mesh] OR “race factors”[Mesh] OR racism[Mesh] OR “African American*”[Title/Abstract] OR “African ancestry”[Title/Abstract] OR AIAN[Title/Abstract] OR “Alaska Nativ*”[Title/Abstract] OR Asian[Title/Abstract] OR Asians[Title/Abstract] OR “Black American*”[Title/Abstract] OR Caucasian*[Title/Abstract] OR “ethnic disparit*”[Title/Abstract] OR “ethnic differen*”[Title/Abstract] OR “ethnic group*”[Title/Abstract] OR “ethnic inequalit*”[Title/Abstract] OR “ethnic inequit*”[Title/Abstract] OR “ethnic population*”[Title/Abstract] OR ghetto*[Title/Abstract] OR Hispanic*[Title/Abstract] OR Indian[Title/Abstract] OR Indians[Title/Abstract] OR Latina[Title/Abstract] OR Latinas[Title/Abstract] OR Latino[Title/Abstract] OR Latinos[Title/Abstract] OR Latinx*[Title/Abstract] OR “Native American*”[Title/Abstract] OR “Native Hawaiian*”[Title/Abstract] OR “Pacific Islander*”[Title/Abstract] OR “people of color”[Title/Abstract] OR “people of colour”[Title/Abstract] OR “race factor*”[Title/Abstract] OR “race and ethnicity”[Title/Abstract] OR “racial and ethnic minorit*”[Title/Abstract] OR “racial discrimination*”[Title/Abstract] OR “racial disparit*”[Title/Abstract] OR “racial differen*”[Title/Abstract] OR “racial equali*”[Title/Abstract] OR “racial equit*”[Title/Abstract] OR “racial inequal*”[Title/Abstract] OR “racial inequi*”[Title/Abstract] OR “racial prejudice*”[Title/Abstract] OR “racial segregation*”[Title/Abstract] OR racism[Title/Abstract] OR slum[Title/Abstract] OR slums[Title/Abstract] OR “black african”[Title/Abstract] OR “ethnic variation*”[Title/Abstract] | 615,749 |
| #13 | #1 AND #12 | 171 |
| Education or literacy | | |
| #14 | “Educational Status”[Mesh] OR “Health Literacy”[Mesh] OR “Educational Status”[Title/Abstract] OR “Educational Achievement*”[Title/Abstract] OR “Educational Attainment*”[Title/Abstract] OR “Educational Level*”[Title/Abstract] OR “Academic Failure*”[Title/Abstract] OR Literacy[Title/Abstract] OR Illiteracy[Title/Abstract] | 131,967 |
| #15 | #1 AND #14 | 5 |
| Gender identity or sex | | |
| #16 | “Sexual and Gender Minorities”[Mesh] OR “gender disparit*”[Title/Abstract] OR “Sexual minorit*”[Title/Abstract] OR “Gender Minorit*”[Title/Abstract] OR “sexual divers*”[Title/Abstract] OR “sexual orientation*”[Title/Abstract] OR “Homosexuality”[Mesh] OR homosexual*[Title/Abstract] OR “homo sexual*”[Title/Abstract] OR gay[Title/Abstract] OR gays[Title/Abstract] OR lesbian*[Title/Abstract] OR “men who have sex with men”[Title/Abstract] OR “men having sex with men”[Title/Abstract] OR “men who have sex with other men”[Title/Abstract] OR “women who have sex with women”[Title/Abstract] OR “women having sex with women”[Title/Abstract] OR “women who have sex with other women”[Title/Abstract] OR homophil*[Title/Abstract] OR “Bisexuality”[Mesh] OR bisexual*[Title/Abstract] OR pansexual*[Title/Abstract] OR polysexual*[Title/Abstract] OR queer*[Title/Abstract] OR asexual*[Title/Abstract] OR “Transsexualism”[Mesh] OR transgender*[Title/Abstract] OR transsexual*[Title/Abstract] OR transman[Title/Abstract] OR transmen[Title/Abstract] OR “trans men”[Title/Abstract] OR transwoman[Title/Abstract] OR “trans women”[Title/Abstract] OR transwomen[Title/Abstract] OR intersex*[Title/Abstract] OR “gender-expansive”[Title/Abstract] OR “gender change*”[Title/Abstract] OR “gender confirmation*”[Title/Abstract] OR “gender disorder*”[Title/Abstract] OR “gender dysphoria*”[Title/Abstract] OR “Gender Identity”[Mesh] OR “gender identit*”[Title/Abstract] OR “gender non conforming”[Title/Abstract] OR “gender nonconforming”[Title/Abstract] OR genderqueer[Title/Abstract] OR “gender reassignment*”[Title/Abstract] OR “sex reassignment*”[Title/Abstract] OR “gender surger*”[Title/Abstract] OR LGBT*[Title/Abstract] OR GLBT*[Title/Abstract] OR LGBBTQ[Title/Abstract] OR “2 spirit*”[Title/Abstract] OR “two spirit*”[Title/Abstract] OR bigender*[Title/Abstract] OR “gender divers*”[Title/Abstract] OR “gender fluid*”[Title/Abstract] OR “non binary”[Title/Abstract] OR nonheterosexual*[Title/Abstract] OR “non heterosexual*”[Title/Abstract] | 116,543 |
| #17 | #1 AND #16 | 2 |
| Racial differences in orbit anatomy | | |
| #18 | (“orbit anatomy”[Title/Abstract:~5] OR “orbit size”[Title/Abstract:~5] OR “orbit shape”[Title/Abstract:~5] OR “orbital anatomy”[Title/Abstract:~5] OR “orbital size”[Title/Abstract:~5] OR “orbital shape”[Title/Abstract:~5]) AND (“racial differen*”[Title/Abstract] OR “racial variation*”[Title/Abstract] OR “racial background*”[Title/Abstract] OR “ethnic differen*”[Title/Abstract] OR “ethnic variation*”[Title/Abstract] OR “ethnic background*”[Title/Abstract] OR “race factor*”[Title/Abstract] OR “ethnic factor*”[Title/Abstract]) | 10 |
| Exclusions | | |
| #19 | “Animals”[Mesh] NOT “Humans”[Mesh] | 5,220,426 |
| #20 | “Comment”[Publication Type] OR “Letter”[Publication Type] OR “Editorial”[Publication Type] OR “Case reports”[Publication type] OR “case stud*”[Title] OR “case report*”[Title] OR “case series”[Title] OR “case histor*”[Title] | 4,537,055 |
| Total | | |
| #21 | (#3 OR #5 OR #7 OR #9 OR #11 OR #13 OR #15 OR #17 OR #18) | 227 |
| #22 | (#3 OR #5 OR #7 OR #9 OR #11 OR #13 OR #15 OR #17 OR #18) NOT (#19 OR #20) | 199 |
| #23 | (#22 AND (English[Language])) AND ((“2019/05/16”[Date - Publication]: “3000”[Date - Publication])) | 66 |

TED = thyroid eye disease.

Note: Search conducted May 2024.
