# Supplementary material for: The burden of illness in thyroid eye disease: current state of the evidence
Source: Front Ophthalmol (Lausanne). 2025 Apr 17;5:1565762. doi: 10.3389/fopht.2025.1565762 (PMC12075187; doi:10.3389/fopht.2025.1565762)
Supplement: Supplementary file 3 [file Table3.docx]

# Supplementary Material

Table S3. Ongoing Clinical Trials on TED Treatments as of May 2024

| Product name, study description | Target of inhibition | Study design | Outcome measure(s) |
| --- | --- | --- | --- |
| Teprotumumab-trbw  [NCT05002998](https://clinicaltrials.gov/ct2/show/NCT05002998): Tepezza (teprotumumab-trbw) postmarketing requirement study | IGF-1R | Phase 3b/4, interventional study | Primary outcomes:   - Percentage of participants who experience at least 1 treatment-emergent adverse event and the percentage of participants who experience at least 1 treatment-emergent AESI during treatment with teprotumumab - Percentage of participants who receive retreatment |
| Teprotumumab-trbw  OPTIC-J (Japan Registry of Clinical Trials ID number [jRCT2031210453](https://jrct.niph.go.jp/en-latest-detail/jRCT2031210453)): A study evaluating Tepezza for the treatment of active TED in Japan | IGF-1R | Phase 3, interventional study | Primary outcome:  Proptosis response rate at week 24, measured as the percentage of participants with ≥2 mm reduction in proptosis from baseline in the study eye without deterioration in the fellow eye (defined as a ≥2 mm increase) |
| VRDN-001  [NCT05176639](https://clinicaltrials.gov/ct2/show/NCT05176639): A safety, tolerability, and efficacy study of veligrotug (VRDN-001) in healthy volunteers and persons with TED | IGF-1R | Phase 2/3, interventional study | Primary outcomes:   - Number of participants with treatment-emergent adverse events - Proptosis responder rate   Secondary outcomes:   - Change from baseline in measurement of proptosis as determined by exophthalmometer - Change from baseline in volume of orbital fat as determined by MRI - Change from baseline in volume of extraocular muscles as determined by MRI - Change from baseline in CAS - Overall Responder Rate comprising Proptosis Responder Rate as measured by exophthalmometer and Clinical Activity Responder Rate in the study eye - Clinical Activity Responder Rate in the study eye - Diplopia responder rate - Diplopia resolution rate - Proportion of participants with a CAS of zero or one in the study eye |
| VRDN-001  [NCT06021054](https://classic.clinicaltrials.gov/ct2/show/NCT06021054): A phase 3, randomized, double-blind study of veligrotug (VRDN-001) in patients with chronic TED | IGF-1R | Phase 3, interventional study | Primary outcomes:  Proptosis responder rate at week 15  Secondary outcomes:   - Change from baseline in proptosis at week 15 - Clinical activity responder rate - Overall response rate - Diplopia resolution rate |
| Lonigutamab  [NCT05683496](https://clinicaltrials.gov/ct2/show/NCT05683496): Efficacy and safety of lonigutamab in subjects with TED | IGF-1R | Phase 1/2, interventional study | Primary outcome:  Incidence and characterization of nonserious and serious treatment-emergent adverse events from day 1-169  Secondary outcomes:   - Pharmacokinetics profile of lonigutamab from day 1-169: - Maximum observed concentration (C_max_) - Minimum observed concentration (C_min_) |
| Linsitinib  [NCT05276063](https://clinicaltrials.gov/ct2/show/NCT05276063): A phase 2b study of linsitinib in subjects with active, moderate-to-severe TED (LIDS) | IGF-1R | Phase 2b, interventional study | Primary outcome:  Percentage of patients who are proptosis responders at week 24  Secondary outcomes:   - Change from baseline in proptosis to week 24 - Percentage of patients who are diplopia responders and overall responders at week 24 - Percentage of patients who are CAS categorical responders at week 24 - Change from baseline in the GO-QOL overall score to week 24 |
| SHR-1314  [NCT05394857](https://clinicaltrials.gov/ct2/show/NCT05394857): Efficacy and safety of vunakizumab (SHR-1314) by subcutaneous injection in active, moderate-to-severe GO patients | IL-17A | Phase 2, interventional study | Primary outcome:  Proptosis responder rate (week 16)  Secondary outcomes:   - Proportion of patients achieving response in reduction of CAS - Overall responder rate at week 16 - Percentage of patients with CAS of 0 or 1 in the study eye - Mean change from baseline to week 16 in proptosis measurement in the study eye - Diplopia response rate at week 16 - Mean change from baseline to week 16 in the GO-QOL score |
| Batoclimab^a^  [NCT05524571](https://clinicaltrials.gov/ct2/show/NCT05524571): Study to assess batoclimab in participants with active TED | FcRn | Phase 3, interventional study | Primary outcome:  Percentage of proptosis responders  Secondary outcomes:   - Percentage of participants with proptosis ≥2 mm reduction and CAS ≤3 from baseline in the study eye - Percentage of participants with CAS of 0 or 1 in the study eye - Mean change from baseline in CAS in the study eye - Percentage of participants with positive binding anti-TSHR antibody at baseline who achieve seroconversion - Percentage of participants with decrease of at least 1 grade from Baseline in Gorman score for diplopia - Mean change from Baseline in proptosis in the study eye - Percentage of participants with ≥6-point increase from Baseline in total Graves' ophthalmopathy - Quality of life (GO-QOL) score - Percentage of participants with ≥8-degree increase from Baseline in motility (in at least 1 of 4 directions) in the study eye |
| Batoclimab^a^  [NCT05517421](https://clinicaltrials.gov/ct2/show/NCT05517421): Study to assess batoclimab in participants with active TED | FcRn | Phase 3, interventional study | Primary outcome:  Percentage of proptosis responders  Secondary outcomes:   - Percentage of participants with proptosis ≥2 mm reduction and CAS ≤3 from baseline in the study eye - Percentage of participants with CAS of 0 or 1 in the study eye - Mean change from baseline in CAS and in proptosis in the study eye - Percentage of participants with positive binding anti-TSHR antibody at baseline who achieve seroconversion - Percentage of participants with a decrease of at least 1 grade from baseline in Gorman score for diplopia - Percentage of participants with ≥6-point increase from baseline in total GO-QOL score - Percentage of participants with ≥8-degree increase from baseline in motility |
| Batoclimab^a^  [NCT05517447](https://clinicaltrials.gov/ct2/show/NCT05517447): Extension study to assess batoclimab in participants with TED | FcRn | Phase 3, interventional study | Primary outcome:  Duration of proptosis response off treatment in the study eye in batoclimab responder participants in the feeder studies  Secondary outcomes:   - Percentage of proptosis in the study eye responders among placebo nonresponder participants in the feeder studies - Percentage of proptosis in the study eye responders among batoclimab nonresponder participants in the feeder studies |
| Batoclimab  [NCT05015127](https://clinicaltrials.gov/ct2/show/NCT05015127): A study to evaluate the efficacy and safety of batoclimab on moderate-to-severe TED | FcRn | Phase 2/3, interventional study | Primary outcome:  Proptosis responder rates of the treatment groups and placebo group at week 12  Secondary outcomes:   - Percentage of patients with CAS of 0 or 1 in the study eye at weeks 6, 12, 18, and 24 - Change from baseline in proptosis measurement in the study eye at weeks 6, 12, 18, and 24 - Composite responder rate at weeks 6, 12, 18, and 24 - Diplopia response rate at weeks 6, 12, 18, and 24 - Change from baseline in the GO-QOL visual function score and psychosocial function score at weeks 6, 12, 18, and 24 |
| Atorvastatin  [NCT05049603](https://clinicaltrials.gov/ct2/show/NCT05049603): A randomized clinical trial to evaluate the effects of atorvastatin on Graves’ orbitopathy (STAGO-2) | HMG-CoA | Phase 3, interventional study | Primary outcome:  Outcome of TED  Secondary outcomes:   - Outcome of TED at 12 and 48 weeks - QOL (comparison between the 2 groups) at 12, 24, and 48 weeks - TED relapse at 24 and 48 weeks |
| Simvastatin  [NCT03131726](https://clinicaltrials.gov/ct2/show/NCT03131726): Treatment of GO with simvastatin (GO-S) | HMG-CoA | Phase 3, interventional study | Primary outcomes:   - Change in CAS after 6 months - Number of patients with progression to severe TED during 6 months   Secondary outcomes:   - Change in modified CAS after 3 and 6 months - Optical coherence tomography after 3 and 6 months - QOL with SF-36, ThyrPro, and GO-QOL after 6 months - TSHR antibodies and TPO antibodies after 3 and 6 months |
| Methimazole  [NCT04776993](https://clinicaltrials.gov/ct2/show/NCT04776993): A conservative vs. an ablative approach for treatment of hyperthyroidism in patients with GO (ABLAGO) | TPO | Phase 3, interventional study | Primary outcome:  Overall TED outcome at 24 weeks; improvement is defined as change in 2 of the following outcome measures in at least 1 eye, without deterioration in any of the same measures in both eyes:   - Improvement in CAS by ≥2 points - Improvement in exophthalmos by ≥2 mm (measured by Hertel exophthalmometer) - Improvement in lid aperture by ≥2 mm - Improvement in diplopia (disappearance or change in degree) - Improvement of visual acuity by ≥0.2/1   Secondary outcomes:   - Overall TED outcome at 48 and 72 weeks - Response of individual TED parameters: proptosis, CAS, eyelid width, diplopia, and visual acuity - GO-QOL at 24, 48, and 72 weeks |
| Glucocorticoids  [NCT04548284](https://clinicaltrials.gov/ct2/show/NCT04548284): Study of therapeutic value of periorbital injection of glucocorticoid in mild thyroid-associated ophthalmopathy | GR | Phase 3, interventional study | Primary outcome:   - Change of CAS from baseline at 3, 6, 9, and 12 months - Change of NOSPECS score from baseline at 3, 6, 9, and 12 months   Secondary outcome:   - Adverse events from baseline and at 3, 6, 9, and 12 months - Change in GO-QOL from baseline at 3, 6, 9, and 12 months |
| Aflibercept  [NCT04311606](https://clinicaltrials.gov/ct2/show/NCT04311606): Anti-VEGF therapy for acute thyroid eye disease (AcTED) | VEGF | Phase 2, interventional study | **Primary outcome:**  Safety and tolerability of sub-Tenon aflibercept in combination with either saline or hyaluronidase in patients with acute TED as assessed by the incidence and severity of adverse events (timeframe: 45 days)  Secondary outcomes:   - Safety and tolerability of sub-Tenon aflibercept in combination with either saline or hyaluronidase in patients with acute TED as assessed by the incidence and severity of adverse events (timeframe: baseline to day 90) - Change in clinical activity as measured by a 2-point decrease on the CAS (timeframe: 1‑90 days) - Change in extraocular muscle diameter as measured by CT scan (timeframe: 1-90 days) - Change in proptosis as measured by Hertel exophthalmometer (timeframe: 1-90 days) |
| Tocilizumab  [NCT04876534](https://clinicaltrials.gov/ct2/show/NCT04876534): Tocilizumab in active moderate-severe GO (TOGO) | IL-6R | Phase 2, interventional study | Primary outcome:  Proportion of patients with CAS reduction of 3 points or disease inactivation (CAS <4) at 12 and 24 weeks  Secondary outcomes:   - Proportion of patients improved at 24 weeks as assessed by the EUGOGO composite ophthalmic score - Improvement of QOL according to the GO-QOL questionnaire at 12 and 24 weeks - Incidence of adverse events in tocilizumab therapy from 0 to 12 weeks - Immunological changes at 12, 24, 36, and 48 weeks of follow-up - Residual disease at 48 weeks - Rehabilitative therapy at 48 weeks |
| TOUR006  [NCT06088979](https://classic.clinicaltrials.gov/ct2/show/NCT06088979): TOUR006 for patients with TED who are in the active inflammatory phase of disease (spiriTED) | IL-6R | Phase 2b, interventional study | Primary outcome:  Percentage of patients achieving a proptosis response defined as a ≥2 mm reduction in proptosis from baseline in the study eye without deterioration  Secondary outcomes:   - Percentage of patients achieving a proptosis response with 20 mg TOUR006 administered subcutaneous every 8 weeks or 50 mg TOUR006 administered subcutaneous every 8 weeks - Percentage of patients attaining a complete or near complete response on the 7-point CAS - Percentage of patients attaining ≥1 grade decrease in diplopia - Incidence of treatment-emergent adverse events by severity and serious adverse events through week 72 - Mean change from baseline in serum trough concentration of TOUR006 - Mean change from baseline in serum TSI (timeframe: 72 weeks) - Percentage of patients with antidrug antibodies. |
| Satralizumab  [NCT05987423](https://classic.clinicaltrials.gov/ct2/show/NCT05987423): Satralizumab in TED patients | IL-6R | Phase 3, interventional study | Primary outcome:  Percentage of participants achieving ≥2 mm reduction in proptosis from baseline (Day 1) at Week 24  Secondary outcomes:   - Change in proptosis - Percentage of participants achieving > = 1 grade reduction/improvement in diplopia among participants with baseline diplopia - Percentage of participants with adverse events, with severity determined according to NCI CTCAE version 5.0 |
| Sirolimus  [NCT04598815](https://clinicaltrials.gov/ct2/show/NCT04598815): Sirolimus for GO (SIRGO) | mTOR | Phase 2, interventional study | Primary outcome:  TED overall response (24 weeks)  Secondary outcomes:   - TED overall response after 12, 36, and 48 weeks - TED relapse after 36 and 48 weeks - Change in exophthalmos after 12, 24, 36, and 48 weeks - Change in CAS after 12, 24, 36, and 48 weeks - Change in GO-QOL after 12, 24, 36, and 48 weeks - Percentage of adverse events at 48 weeks |
| Sirolimus  [EudraCT2021-000641-41](https://www.clinicaltrialsregister.eu/ctr-search/trial/2021-000641-41/NO): Sirolimus vs. corticosteroids in treatment of TED | mTOR | Phase 2 | Primary outcome:  ≥2-point reduction in CAS from baseline at week 12  Secondary outcomes:   - ≥2 mm reduction from baseline in proptosis in 1 eye at week 12 - ≥2 mm reduction from baseline in vertical lid aperture in 1 eye at week 12 - ≥1 class improvement of eye motility from baseline assessed by Gorman score at week 12 - Participants with minor and serious adverse effects after 6 months - ≥6-point improvement in GO-QOL score |
| Doxycycline hyclate  [NCT02203682](https://clinicaltrials.gov/ct2/show/NCT02203682): Doxycycline treatment in mild thyroid-associated ophthalmopathy | 30S prokaryotic ribosomal subunit | Phase 2, interventional study | Primary outcome:  Rate of improvement over 12 weeks, defined as ≥1 item was met in the study eye without deterioration in any item of both eyes:   - Reduction of eyelid aperture by ≥2 mm - Reduction of exophthalmos by ≥2 mm - Increase in ocular motility by ≥8 degrees in any duction - Increase on either GO-QOL scales by ≥6 points   Secondary outcomes:   - CAS at 4 weeks and 12 weeks - Proptosis measured by Hertel instrument at 4 and 12 weeks - Eyelid aperture at 4 and 12 weeks - Lid retraction at 4 and 12 weeks - Lid lag at 4 and 12 weeks - Eye motility at 4 and 12 weeks - GO-QOL at 4 and 12 weeks - C-OSDI at 4 and 12 weeks - Category of adverse events and frequency of occurrence of adverse events at 4 and 12 weeks |
| Rituximab  [EudraCT2012-001980-53](https://www.clinicaltrialsregister.eu/ctr-search/trial/2012-001980-53/IT): Preliminary study on the effectiveness of low doses of rituximab in patients with GO poorly responding to immunosuppressive steroid treatment | CD20 | Phase 3 | Primary outcome:  Reduction of CAS ≥2 points or below CAS = 3  Secondary outcomes:   - Reduction of severity of TED of ≥2 classes per NOSPECS - Reduction of proptosis ≥2 mm - Reduction of lid fissure of ≥2 mm - Reduction of diplopia according to Gorman score ≥1 class or of the motility of ≥8 degrees - Number of therapeutic responses - Number of active disease recurrences - Signs of residual disease - Improvement of GO-QOL scale of ≥6 points   Observational endpoint:  At week 24 to evaluate disease residual and relapses |
| Belimumab  [EudraCT2015-002127-26](https://www.clinicaltrialsregister.eu/ctr-search/trial/2015-002127-26/IT): Comparison between treatment with belimumab and methylprednisolone in Graves’ hyperthyroidism and active orbitopathy | BLyS | Phase 2 | Primary outcomes:   - Decrease of the CAS of 2 points or disease inactivation (CAS <4) in active patients with TED at 12, 24, 36, and 48 weeks from belimumab or methylprednisolone administration - Safety of belimumab therapy in patients with GD and TED   Secondary outcomes:   - Decrease of TED severity by NOSPECS classes 2, 3, or 4 of ≥1 point - Analysis of the rate of response to therapy and of relapse of active disease at 12, 24, 36, and 48 weeks - Time (months) to remission of hyperthyroidism and decrease or absence of serum TSHR antibodies in patients with hyperthyroidism GD at 12, 24, 36, and 48 weeks - Quantification of signs of residual motility abnormalities by motility tests and analysis of GO-QOL - Analysis of intrathyroidal and orbital tissue and lymphocyte BAFF expression after therapy by immunohistochemistry and cytofluorimetry |

AESI = adverse event of special interest; ALT = alanine aminotransferase; AST = aspartate aminotransferase; BAFF = B-cell–activating factor; BLyS = B-lymphocyte stimulator; C_max_ = maximum observed concentration; C_min_= minimum observed concentration; CAS = clinical activity score; CD20 = cluster of differentiation 20; C-OSDI = Chinese Ocular Surface Disease Index; COVID-19 = coronavirus disease 2019; CPK = creatine phosphokinase; CT = computed tomography; EUGOGO = European Group on Graves’ Orbitopathy; FcRn = neonatal Fc receptor; GD = Graves’ disease; GO = Graves orbitopathy; GO‑QOL = Graves’ Ophthalmopathy Quality-of-Life Questionnaire; GR = glucocorticoid receptor; HMG-CoA = 3-hydroxy-3-methylglutaryl-coenzyme A reductase; IGF-1R = insulin-like growth factor 1 receptor; IL-6 = interleukin-6; IL-6R = interleukin-6 receptor; IL-17A = interleukin-17A; IV = intravenous; MAD = multiple ascending dose; MRI = magnetic resonance imaging; mTOR = mechanistic target of rapamycin; NCI CTCAE = National Cancer Institute’s Common Terminology Criteria for Adverse Events; NOSPECS = No signs or symptoms, Only signs/no symptoms, Soft tissue involvement, Proptosis, Extraocular muscle involvement, Corneal involvement, Sight loss; Q2W = every 2 weeks; QOL = quality of life; QW = every week; SF-36 = Short Form Health Survey, 36-Items; TED = thyroid eye disease; ThyrPro = thyroid-related quality-of-life questionnaire; TPO = thyroid peroxidase; TSHR = thyrotropin receptor; VEGF = vascular endothelial growth factor.

Note: Study phase presented as of May 2024, the time the literature review was completed.

^a^ Batoclimab is the first FcRn inhibitor to be investigated in this disease.
